# Sex hormone binding globulin as a valuable biochemical marker in predicting gestational diabetes mellitus

**DOI:** 10.1186/s12905-017-0373-3

**Published:** 2017-03-09

**Authors:** Manal Abdalla Tawfeek, Eman Mohamad Alfadhli, Abdulfatah Marawan Alayoubi, Hesham Ahmad El-Beshbishy, Fawzia Ahmad Habib

**Affiliations:** 1 Clinical Biochemistry and Molecular Medicine Department, Taibahu University, College of Medicine, Madina, Saudi Arabia; 2 Clinical and Chemical Pathology Department, Tanta University, College of Medicine, Tanta, Egypt; 3 Internal Medicine Department, College of Medicine, Taibahu University, Madina, Saudi Arabia; 4 Center for Genetics and Inherited Diseases and Medical Laboratories Technology Department, Taibahu University, Madina, Saudi Arabia; 5 Biochemistry Department, Faculty of Pharmacy, Al-Azhar University, Cairo, Egypt; 6 Obstetrics & Gynecology Department, Taibahu University, College of Medicine, Madina, Saudi Arabia

**Keywords:** Sex hormone binding globulin, Gestational diabetes mellitus, Saudi Arabia, Pregnancy

## Abstract

**Background:**

Circulating Sex hormone binding globulin (SHBG) levels are inversely associated with insulin resistance. This study was conducted to compare maternal serum SHBG level between pregnant women with normal glucose tolerance and those with gestational diabetes (GDM) and to investigate the roll of SHBG in GDM diagnosis.

**Methods:**

This was a case controlled study of 90 pregnant women, 45 women with GDM and 45 matched controls, attending obstetrics clinic at Ohud Hospital, Madina, Saudi Arabia between April 2014 and March 2015. Measurement of serum SHBG levels by Enzyme-linked immunosorbent assay (ELISA) method were done between 24 and 28 weeks of gestation. The best cut-off point of SHBG to diagnose GDM was calculated in receiver operating characteristic curve.

**Results:**

Compared with the control group, SHBG concentrations were significantly lower in the GDM group; median 23 nmol/L (18–30) vs. 78 nmol/L (65–96), *p* < 0.001). The cut off value 50 nmol/L of the SHBG had 90% sensitivity and 96% specificity to diagnose GDM.

**Conclusion:**

Patients with GDM have lower circulating levels of SHBG than normal glucose tolerance pregnant women. Circulating concentrations of SHBG represent a potentially useful new biomarker for prediction of risk of GDM beyond the currently established clinical and demographic risk factors.

## Background

Sex hormone-binding globulin (SHBG) is a glycoprotein produced by the liver that binds sex steroids in the circulation. Secretion is suppressed by insulin, and low levels of SHBG are frequently observed in states of insulin resistance and have been studied as a potential predictor of the development of T2DM [1, 2].

Insulin resistance is the hallmark of gestational diabetes mellitus (GDM) and it is pathogenically related to T2DM. SHBG levels were reported to be lower in women with gestational diabetes and in those who require insulin therapy [3–7]. In addition, women with a reduced concentration of SHBG in the first trimester of pregnancy are at increased risk of developing gestational diabetes later in pregnancy [7–9].

GDM is a common pregnancy complication and is associated with increased maternal and neonatal morbidity. Identifying and treating women with GDM is important to improve the outcomes. The definitive diagnostic testing for GDM is an oral glucose tolerance test. Such test requires fasting of at least 8 h, needs 3–4 blood samplings and requires 2–3 h to be completed. SHBG is simple, inexpensive blood test that can be performed in the non-fasting state, [10] with no diurnal variation, [11]. This makes SHBG a valuable marker for GDM diagnosis. The aim of this study is to compare maternal serum SHBG level between GDM and normal glucose tolerant women and to investigate the roll of SHBG in GDM diagnosis.

## Methods

The study was a hospital based case control study that involved pregnant women followed at the antenatal service of the outpatient clinic at Ohud Hospital, Madina, Saudi Arabia between April 2014 and March 2015. A total of 55 pregnant women with GDM were enrolled in the study between 24 and 28 weeks’ of gestation. The diagnosis of GDM in Ohud hospital is based on the international Association of Diabetes and Pregnancy Study Groups (IADPSG) consensus panel [12]. Women with pre-gestational diabetes mellitus, preeclampsia or gestational/chronic hypertension (systolic blood pressure >140 mmHg and diastolic blood pressure >90 mmHg), multiple pregnancies and patients with poly cystic ovary syndrome (PCOS) were excluded from the study. After the exclusion of ten women, forty five women with GDM were included in the study and were matched with 45 pregnant controls of similar age, weight, height and body mass index(BMI).

Maternal age, weight, height, BMI and blood pressure were recorded. Blood samples were collected from the participants at 24–28 weeks’ of gestation into non- heparinized tubes. Then the blood sample was centrifuged at 3000 rpm for 10 min within 20 min of the blood draw and then separated and divided into two samples, one for analysis of the biochemical parameters and the other was frozen at -20 C until assayed for SHBG analyses.

For the measurement of biochemical parameters the sample was placed on ice and transported to the clinical laboratory in a cooler with an ice block within 2–4 h of being drawn, and the serum glucose concentration was measured in mg/dl by the glucose oxidase method and serum cholesterol concentration was measured by colorimetric method. SHBG was measured by a quantitative sandwich enzyme-linked immunoassay (ELISA) technique. The kit for SHBG analysis was supplied by DIA source Immuno Assays SA- Rue du Bosquet 2, B-1348 Louvain-la -Neuve, Belgium. The analytical sensitivity of the DIA source ELISA was found to be 0.77 nmol/L.

### Statistical analyses

Data analysis was performed using SPSS for Windows, version 20. Whether the distributions of continuous variables were normal or not was determined by the Shapiro–Wilk test. Data are shown as mean ± SD or median (Interquartile range), where applicable. The mean differences between groups were compared by Student’s *t*-test; otherwise the Mann–Whitney *U*-test was applied for the comparisons of the median values. Degrees of association between continuous variables were calculated by Spearman’s rank correlation analyses.

The ability of SHBG value to detect GDM was examined by the receiver operating characteristic (ROC) curve and their respective areas under the curve, in which sensitivity is plotted as a function of 1-specificity. The optimal cut-off points with highest sensitivity and specificity were evaluated. A *P*-value less than 0.05 was considered statistically significant.

## Results

A total of 90 pregnant women were included in the study, 45 women with GDM and 45 matched controls (Non-GDM). Maternal baseline characteristics are shown in (Table 1). There were no significant differences between the two groups with regards to age, weight, height, BMI, and blood pressure.

**Table 1:** Baseline characteristics of the studied groups

Parameters (mean ± SD)	GDM (*n* = 45)	Non-GDM (*n* = 45)	*p* value
Age (years)	29.27 ± 6.87	26.84 ± 6.99	0.101
Weight (Kg)	72.84 ± 8.79	70.84 ± 8.39	0.273
Height (cm)	158.42 ± 6.31	159.40 ± 5.15	0.423
BMI (Kg/m^2^)	30.73 ± 4.82	29.56 ± 3.66	0.198
SBP (mmHg)	121.64 ± 6.28	120.33 ± 6.25	0.324
DBP (mmHg)	73.42 ± 2.83	72.82 ± 2.57	0.295
Serum Cholesterol (mg/dl)	174.56 ± 12.71	158.71 ± 8.96	0.001
RBG (mg/dl)	93.24 ± 4.64	83.91 ± 4.12	0.001

**Fig. 1:**
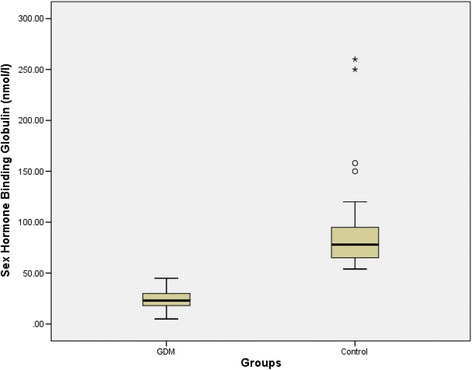
Difference in the median sex hormone binding globulin levels between gestational diabetes mellitus and the control groups

**Fig. 2:**
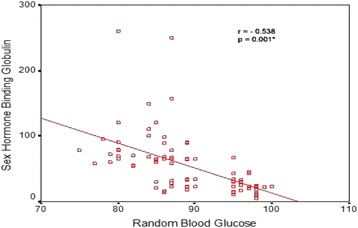
Correlation between random blood glucose levels and sex hormone binding globulin levels in the studied groups

Women with GDM had higher levels of random blood glucose(RBG) and cholesterol than the control group, *P* = 0.001 (Table 1). SHBG concentration was found to be significantly lower in the GDM group; median 23 nmol/L (18–30) than in control group 78 nmol/L (65–96), *P* = 0.001, (Fig. 1). There was significant negative correlation between the levels of SHBG and RBG and cholesterol levels (Table 2 & Fig. 2).

**Table 2:** Correlations between maternal parameters and SHBG in the studied groups

Serum SHBG						
Maternal arameters	GDM group (*n* = 45)		Control group (*n* = 45)		All cases (*n* = 90)	
	r	*p*	r	*p*	R	*p*
Age	0.075	0.626	- 0.019	0.899	- 0.128	0.228
BMI	0.253	0.094	- 0.101	0.508	- 0.112	0.291
RBG	- 0.126	0.409	0.008	0.961	- 0.538	0.001*
Serum cholesterol	-0.210	0.166	- 0.320	0.032*	- 0.547	0.001*

*p value is significant

**Fig. 3:**
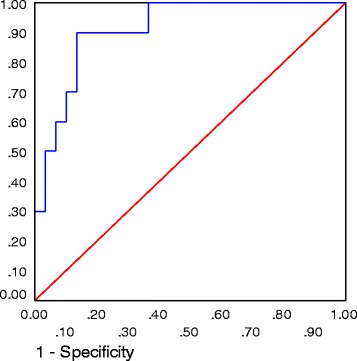
Receiver operator curve shows the predictive probabilities of sex hormone binding globulin levels for gestational diabetes mellitus

The predictive accuracy of SHBG as a marker for GDM was determined by receiver operator curve (ROC) analysis (AUC: 0.913; 95% CI: 0.822-1.005). The cutoff value 50 nmol/L of the SHBG had 90%, sensitivity, 96% specificity, 95% positive predictive values and 89% negative predictive values, (Fig. 3).

## Discussion

SHBG is important for the transport and regulation of sex hormones. It is secreted in the liver under hormonal and nutritional control. Secretion is suppressed by insulin, and low levels of SHBG are frequently observed in states of insulin resistance and have been studied as a potential predictor of the development of T2DM [1, 2]. In normal pregnancy, SHBG levels rise progressively until 24 weeks of gestation [13, 14]. Subsequently, the level of SHBG stabilizes and this may be attributable to the hyperinsulinemia and insulin resistance that increase progressively from the late second trimester [15, 16]. GDM is a state of insulin resistance in pregnancy that seems to result from similar mechanisms in type 2 diabetes mellitus. Sex hormone-binding globulin has emerged as one of the biochemical marker for GDM diagnosis [3–9].

In the current study, we found women with GDM had significantly lower levels of SHBG concentrations compared to Non GDM women at 24–28 weeks of pregnancy. This finding is consistent with results from previous studies [3–9]. Furthermore, lower first-trimester SHBG levels were found to predict subsequent gestational diabetes mellitus [7–9]. Moreover, SHBG were reported to be lower in women with GDM requiring insulin compared to those with medical nutritional therapy alone. On the basis of these results, it was suggested measuring SHBG early in gestation could have a potential benefit in prediction of severe GDM [7]. This might overcome the limitation of the current recommendation for GDM diagnosis which recommend screening at 24 to 28 weeks of gestation that leaves a narrow window during which interventions can be applied before delivery. Earlier identification and treatment of pregnancies with, or at risk for, GDM with SHBG might present a good option to improve outcomes. On the same manner, preconception SHBG levels in women with PCOS were reported to be strongly associated with subsequent development of GDM. PCOS is associated with insulin resistance which will be augmented by the hormones of pregnancy that counter the action of insulin. Therefore, measuring SHBG pre conception was suggested to be a screening tool of women at higher risk of developing GDM during pregnancy [17]. Furthermore, lower SHBG levels were reported to be associated with higher fasting blood glucose levels among women with recent GDM, a high-risk population for diabetes, and this association was independent of potential confounders [18]. Thus, SHBG might be a useful marker in predicting T2DM development in women with recent GDM, however, this requires further testing.

## Conclusion

Patients with GDM have a lower circulating level of SHBG than normal glucose tolerance pregnant women. Circulating concentrations of SHBG represent a potentially useful new biomarker identifying GDM beyond the currently established biochemical markers. A standard assay for serum SHBG analyses and a gestational trimester threshold level have to be determined.

## Abbreviations

AUC: Area under the curve
BMI: Body mass index
CI: Confidence interval
DBP: Diastolic blood pressure
GDM: Gestational diabetes mellitus
PCOS: Poly cystic ovary syndrome
RBG: Random blood glucose
RBG: Random blood glucose
ROC: Receiver operating characteristic curve
SBP: Systolic blood pressure
SD: Standard deviation
SHBG: Sex hormone binding globulin
T2DM: Type 2 diabetes mellitus
WHO: World health organization
